# *In vitro *and *in vivo *evaluation of NCX 4040 cytotoxic activity in human colon cancer cell lines

**DOI:** 10.1186/1479-5876-3-7

**Published:** 2005-02-03

**Authors:** Anna Tesei, Paola Ulivi, Francesco Fabbri, Marco Rosetti, Carlo Leonetti, Marco Scarsella, Gabriella Zupi, Dino Amadori, Manlio Bolla, Wainer Zoli

**Affiliations:** 1 Division of Oncology and Diagnostics, Morgagni-Pierantoni Hospital, Forlì, Italy; 2 Istituto Oncologico Romagnolo, Forlì, Italy; 3 Preclinical Experimental Laboratory, Regina Elena Institute for Cancer Research, Rome, Italy; 4 NicOx SA, Sophia-Antipolis, France

## Abstract

**Background:**

Nitric oxide-releasing nonsteroidal antiinflammatory drugs (NO-NSAIDs) are reported to be safer than NSAIDs because of their lower gastric toxicity. We compared the effect of a novel NO-releasing derivate, NCX 4040, with that of aspirin and its denitrated analog, NCX 4042, in *in vitro *and *in vivo *human colon cancer models and investigated the mechanisms of action underlying its antitumor activity.

**Methods:**

*In vitro *cytotoxicity was evaluated on a panel of colon cancer lines (LoVo, LoVo Dx, WiDr and LRWZ) by sulforhodamine B assay. Cell cycle perturbations and apoptosis were evaluated by flow cytometry. Protein expression was detected by Western blot. In the *in vivo *experiments, tumor-bearing mice were treated with NCX 4040, five times a week, for six consecutive weeks.

**Results:**

In the *in vitro *studies, aspirin and NCX 4042 did not induce an effect on any of the cell lines, whereas NCX 4040 produced a marked cytostatic dose-related effect, indicating a pivotal role of the -NO_2 _group. Furthermore, in LoVo and LRWZ cell lines, we observed caspase-9 and -3-mediated apoptosis, whereas no apoptotic effect was observed after drug exposure in WiDr or LoVo Dx cell lines. In *in vivo *studies, both NCX 4040 and its parental compound were administered *per os*. NCX 4040 induced a 40% reduction in tumor weight. Conversely, aspirin did not influence tumor growth at all.

**Conclusions:**

NCX 4040, but not its parental compound, aspirin, showed an *in vitro *and *in vivo *antiproliferative activity, indicating its potential usefulness to treat colon cancer.

## Background

One of the most important approaches for reducing cancer incidence is chemoprevention. This is especially relevant for colon cancer, which represents the third leading cause of cancer mortality in developing countries and for which diagnostic tests and clinical treatments are not satisfactory [1].

Recently published reviews [2-4] underline the growing role of nonsteroidal antiinflammatory drugs (NSAIDs) in preventing colon cancer. Epidemiologic studies [5] have found that long-term users of aspirin or other NSAIDs have a lower risk of colorectal adenomatous polyps and colorectal cancer compared to nonusers. Randomized clinical trials have confirmed that two NSAIDs, sulindac [6-9] and the selective cyclooxygenase (COX)-2 inhibitor, celecoxib [10], effectively inhibit the growth of adenomatous polyps and cause regression of existing polyps in patients with familial adenomatous polyposis (FAP), although a recent study showed very clear benefits only for the first six months of treatment with sulindac [11].

Despite a multiplicity of studies conducted on this compound family of drugs, little is known about the molecular targets that are responsible for their tumor-preventing properties and which are important, not only from a mechanistic point of view, but also because of their clinical implications in identifying individuals or subsets of patients who are sensitive to these drugs. Originally, it was believed that the regular use of these compounds acted exclusively through the inhibition of COX-1 and COX-2 activity involved in the production of prostaglandins [12]. There is now mounting evidence that the chemopreventive action of NSAIDs may involve COX-independent mechanisms [13,14]. Furthermore, despite initial enthusiasm about the potential relevance of NSAIDs, especially the selective COX-2 inhibitors, their use as chemopreventive or anticancer agents has been greatly limited by side-effects on gastrointestinal and renal systems.

In an attempt to reduce gastrointestinal toxicity, conventional NSAIDs have been coupled with a nitric oxide (NO)-releasing moiety [15,16]. The rationale for this approach is that NO compensates for the functions of prostaglandins, which are inhibited by conventional NSAIDs, in the gastrointestinal tract. Both prostaglandins and NO are capable of enhancing mucosal blood flow, mucus release and repair of mucosal injury in humans [17-19] and of reducing the severity of gastric injury in experimental models [16].

Interestingly, the activity of some NO-NSAIDs is considerably higher than that of the parental drugs. For example, NCX 4016 has been found to be much more effective than the parent aspirin in reducing aberrant crypt foci, a precancerous lesion, in a rat model of colorectal cancer [20] and in inducing cell perturbations and growth inhibition in a panel of colorectal cancer cell lines *in vitro *[21]. Similarly, Williams et al. [22,23], in another panel of human colon cancer lines, reported a higher antiproliferative activity of NO-NSAID compounds compared to the parent aspirin.

In the present work, we compared the effect of a novel NO-releasing derivate, NCX 4040, with that of aspirin and its denitrated analog in *in vitro *and *in vivo *human colon cancer models and investigated the mechanisms of action underlying its antitumor activity.

## Materials and methods

### In vitro studies

#### Cell lines

The studies were performed on colon adenocarcinoma cell lines: 2 (LoVo and WiDr) were obtained from the American Type Culture Collection (Rockville, MD), 1 (LRWZ) was isolated in our laboratory and derived from a patient with a confirmed diagnosis of colon adenocarcinoma, and 1 was doxorubicin-resistant (LoVo Dx) derived from the above-mentioned LoVo cells. Cell lines were maintained as a monolayer at 37°C and subcultured weekly. Culture medium was composed of DMEM/HAM F12 (1:1) supplemented with fetal calf serum (10%), glutamine (2 mM), non-essential aminoacids (1%) (Mascia Brunelli s.p.a., Milan, Italy), and insulin (10 μg/ml) (Sigma Aldrich, Milan, Italy). Cells were used in the exponential growth phase in all the experiments.

#### Drugs

**Figure 1:**
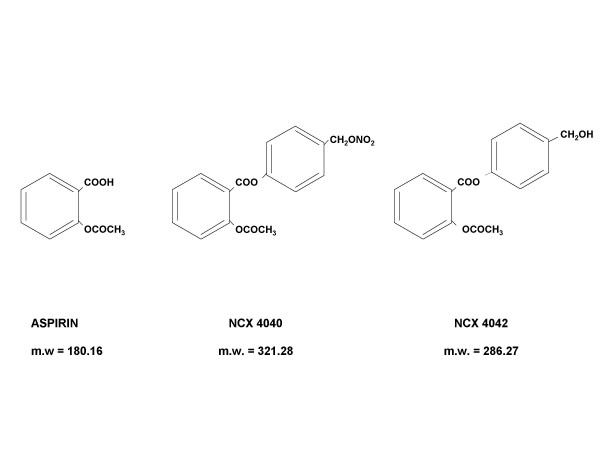
Chemical structure and molecular weight (m.w.) of traditional aspirin, its NO-derivative, NCX 4040, and the NCX 4040 denitrated analog, NCX 4042.

Aspirin, NCX 4040 (NO-releasing aspirin), NCX 4042 (denitrated analog of NCX 4040) (Fig. 1), (all supplied by NicOx S.A., Sophia Antipolis, France), Z-LEHD-FMK (caspase-9 inhibitor) (BD Biosciences Pharmingen, Milan, Italy), sodium nitroprusside dihydrate and S-nitroso-N-acetylpenicillamine (NO donors) (Sigma Aldrich) were solubilized in dimethylsulfoxide (DMSO) (Sigma Aldrich) and freshly diluted in culture medium before each experiment. The final DMSO concentration never exceeded 1% and this condition was used as control in each experiment.

#### Chemosensitivity assay

Sulforhodamine B (SRB) assay was used according to the method by Skehan et al [24]. Briefly, cells were collected by trypsinization, counted and plated at a density of 10,000 cells/well in 96-well flat-bottomed microtiter plates (100 μl of cell suspension/well). In the chemosensitivity assay, NCX 4040, NCX 4042 and aspirin were tested at scalar concentrations ranging from 1 to 100 μM for 24 h and 48 h or for 24 h and 48 h followed by a 24-h culture in drug-free medium. Experiments were run in octuplet, and each experiment was repeated three times. The optical density of treated cells was determined at a wavelength of 540 nm by means of a fluorescence plate reader. Growth inhibition and cytocidal effect of drugs were calculated according to the formula reported by Monks et al. [25]: [(ODtreated - ODzero)/(ODcontrol - ODzero)] × 100%, when ODtreated is > to ODzero. If ODtreated is below ODzero, cell killing has occurred. The ODzero depicts the cell number at the moment of drug addition, the ODcontrol reflects the cell number in untreated wells and the ODtreated reflects the cell number in treated wells on the day of the assay.

#### Western blot

The cells were treated according to the previously described Western blot procedure. [26] COX-1 antibody (polyclonal C-20, dilution 1:100) was purchased from Santa Cruz-Biotechnology (Santa Cruz, CA), COX-2 (monoclonal C22420, dilution 1:250) from Transduction Laboratories (Lexington, KY), and caspases-3 and -9 (polyclonal antibodies, dilution 1:500) from Cell Signaling Technology, Inc. (Beverly, MA).

#### RT-PCR

Total RNA was isolated from cells by direct lysis and quantified spectrophotometrically. After quantification, RT reaction was performed with 1 μg of each sample using Gene Amp Gold RNA PCR Core Kit (Perkin Elmer Biosystems, Milan, Italy). The same cDNA was used for RT-PCR amplification of COX-1 and COX-2. PCR reactions were carried out in a final volume of 25 μl containing 2 μl of cDNA template, 1 unit of Klen Taq, 0.2 mM dNTP and 0.4-μM amounts of each forward and reverse primer, using a thermal cycler (PTC 200, Genenco, Florence, Italy). 5-μl aliquots of the amplified DNA fragments were separated on an ethidium bromide-stained 2% agarose gel.

#### Cell cycle distribution

After a 24-h exposure to 10 μM of NCX 4040, cells were harvested and stained in a solution containing RNase (10 Kunits/ml; Sigma Aldrich) and NP40 (0.01%; Sigma Aldrich). After 30–60 min, samples were analyzed by flow cytometry using a FACS Vantage flow cytometer. Data acquisition (10,000 events were collected for each sample) was performed using CELLQuest software. Data were elaborated using Modfit (DNA Modelling System) software and expressed as fractions of cells in the different cycle phases. Samples were run in triplicate, and each experiment was repeated three times.

#### Apoptosis

Apoptosis was evaluated by flow cytometric analysis according to the previously described TUNEL assay procedure [26]. Briefly, after a 24- and 48-h exposure to 5, 10 and 50 μM of NCX 4040, cells were trypsinized, fixed, exposed to TUNEL reaction mixture and counterstained with propidium iodide before FACS analysis.

In LoVo cells, positivity to TUNEL assay was also evaluated by fluorescence photomicroscope (Zeiss, Axioscope 40) according to the manufacturer's instructions (*In situ *cell death detection kit, fluorescein; Roche Diagnostic GmbH, Mannheim, Germany).

Finally, the cell-permeable DNA dye 4',6-DAPI and a fluorescence photomicroscope (Zeiss, Axioscope 40) were used to visualize chromatin condensation and/or fragmentation typical of apoptotic cells.

### In vivo studies

#### Animals

Antitumor efficacy was evaluated on 6–8-week old CD-1 male nude (nu/nu) mice weighing 22–24 g (Charles River Laboratories, Calco, Italy). All procedures involving animals and their care were conducted in conformity with institutional guidelines, which are in compliance with national (D.L. No. 116, G.U., Suppl. 40, Feb. 18, 1992; Circolare No. 8, G.U., July 1994) and international laws (EEC Council Directive 86/609, OJ L 358. 1, Dec 12, 1987; Guide for the Care and Use of Laboratory Animals, United States National Research Council, 1996).

#### Drugs

NCX 4040 and aspirin were homogeneously suspended in 0.5% carboxymethyl cellulose (CMC) containing 10% of DMSO for antitumor efficacy studies. All the drugs were administered orally.

#### Antitumor efficacy

Since LRWZ line showed a low tumorigenicity when cells were injected into nude mice, *in vivo *experiments were performed on WiDr, LoVo and LoVo Dx lines. Tumor cells were resuspended (5 × 10^6 ^viable cells) in 0.2 ml of serum-free medium and injected into the hind leg muscles of mice.

Each experimental group included at least 6 mice. 10 mg/kg of aspirin and NCX 4040 were administered orally five days a week for 6 consecutive weeks starting on the 6^th ^day after the tumor cell implant, when a tumor mass of about 300 mg was evident in all the animals. The dose of aspirin and NCX 4040 was chosen on the basis of previous observations obtained using the parental NCX 4016 compound [27]. Toxicity of treatments was evaluated in terms of body weight loss and drug deaths. The tumor weight was calculated from caliper measurements according to the method of Geran et al. as previously reported [28]. The antitumor efficacy of treatments was assessed by the following endpoints: a) percent tumor weight inhibition (TWI%), calculated as [1-(mean tumor weight of treated mice/mean tumor weight of controls)] × 100; b) tumor growth delay, evaluated as *T – C*, where *T *and *C *are the median times for treated and control tumors, respectively, to achieve equivalent size.

The significance of results was analyzed by the Mann-Whitney non parametric test. Differences were considered significant at P values < 0.05 (two-sided).

## Results

### In vitro studies

**Figure 2:**
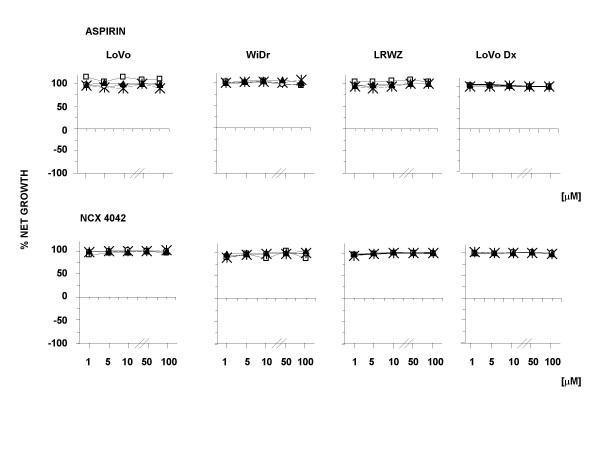
Antiproliferative and cytocidal activity of aspirin and NCX 4042 at concentrations of 1, 5, 10, 50 and 100 μM. Exposure time to drugs:□, 24 h; Ж, 24 h + 24-h washout; ○, 48 h; ▲, 48 h + 24-h washout. Each point indicates the mean of at least three experiments and SD never exceeded 5%.

**Figure 3:**
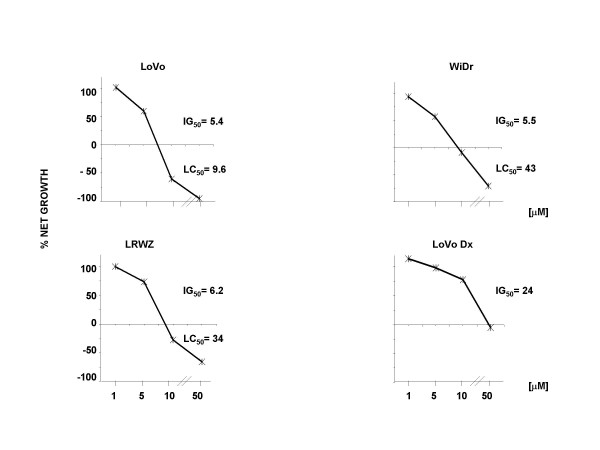
Cytotoxic activity of NCX 4040 after 24-h exposure followed by 24-h washout. Ж, 24 h + 24 h-washout. The two drugs were used at concentrations of 1, 5, 10 and 50 μM. Each point indicates the mean of at least three experiments; SD never exceeded 5%.

The effect of aspirin, NCX 4040, and its structural analog, NCX 4042, on cell growth was determined after different exposure schemes and using various concentrations. Aspirin and NCX 4042 did not exhibit an effect on any of the cell lines (Fig. 2). In contrast, NCX 4040 showed both cytostatic and cytocidal effects, as evaluated by Monk's model (Fig. 3). In particular, the highest cytostatic effect was observed after a 24-h drug exposure followed by a 24-h washout, with a 50% growth inhibition (GI_50_) ranging from 5.4 μM in LoVo cells to 24 μM in LoVo Dx. The same treatment scheme also induced the highest cytocidal effect, with a modulation for the different cell lines and a 50% lethal concentration (LC_50_) in LoVo cells (9.6 μM) about four- and threefold lower than that observed in WiDr and LRWZ, respectively. In LoVo Dx, no cytocidal effect was observed at any of the concentrations tested.

Flow cytometric analysis of apoptosis performed after exposure to various concentrations of NCX 4040 showed 90% of apoptotic cells at 24 h starting from a 10-μM concentration in LoVo line, whereas apoptosis was not detected at the same time in any other cell line at any of the concentrations tested (Table 1). After a 48-h exposure, apoptosis was also induced in LRWZ cells, albeit to a lesser degree, at 10-μM (20%) and 50-μM (60%) concentrations.

**Table 1:** Apoptotic cells after a 24- or 48-h exposure to various concentrations of NCX 4040

**Cell Line**	**24 h**			**48 h**		
		
	**5 μM**	**10 μM**	**50 μM**	**5 μM**	**10 μM**	**50 μM**
**LoVo Dx**	0.7 ± 0.01	0.5 ± 0.01	0.5 ± 0.02	0.5 ± 0.0	0.02 ± 0.01	0.2 ± 0.0
**WiDr**	1.0 ± 0.01	4.0 ± 0.1	1.0 ± 0.05	2.0 ± 0.03	5.0 ± 0.02	5.0 ± 0.03
**LRWZ**	3.0 ± 0.03	5.0 ± 0.02	4.0 ± 0.1	4.0 ± 0.01	21.0 ± 0.3	60.0 ± 1.3
**LoVo**	2.0 ± 0.05	90.0 ± 2.3	n.e.*	3.0 ± 0.02	n.e.	n.e.

* n.e.: not evaluated

**Figure 4:**
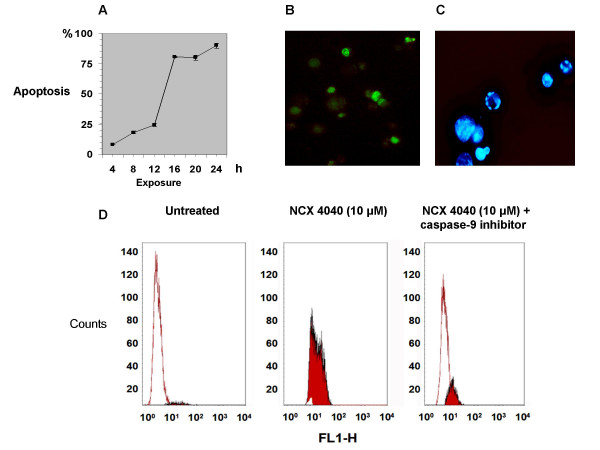
(A) Percentage of apoptosis in LoVo cells after exposure times of 4, 8 12, 16, 20 and 24 h to 10 μM of NCX 4040. (B) Apoptosis in LoVo cells after a 24-h exposure as evidenced by *in situ *TUNEL assay. (C) Apoptosis in LoVo cells after a 24-h exposure as evidenced by DAPI staining. (D) Inhibition of NCX 4040-induced apoptosis in LoVo cells after a 24-h simultaneous exposure to the NO-NSAID and the caspase-9 inhibitor, as evidenced by TUNEL assay.

In LoVo cells, which proved to be the biological system most sensitive to NO-aspirin, the induction of apoptosis produced by a 10-μM concentration was analyzed as a function of time. The results showed that the production of apoptotic cells is time-dependent, reaching as much as 80% after a 16-h drug exposure (Fig. 4A). Apoptotic elements were clearly visible using the *in situ *TUNEL analysis (Fig. 4B) and at fluorescent microscopic examination of cells stained with DAPI nuclear dye (Fig. 4C). Furthermore, cell death was strongly inhibited by simultaneous exposure of LoVo cells to a 10-μM concentration of NCX 4040 and a 100-μM concentration of caspase-9 inhibitor (Fig. 4D). Similar findings were obtained after exposure to caspase-3 inhibitor (data not shown).

To understand the mechanisms responsible for the induction or lack of apoptosis in the different cell lines, we explored the modulation of COX-2 expression and the activation of caspases-3 and -9, which are involved in the mitochondrial pathway of apoptosis.

**Figure 5:**
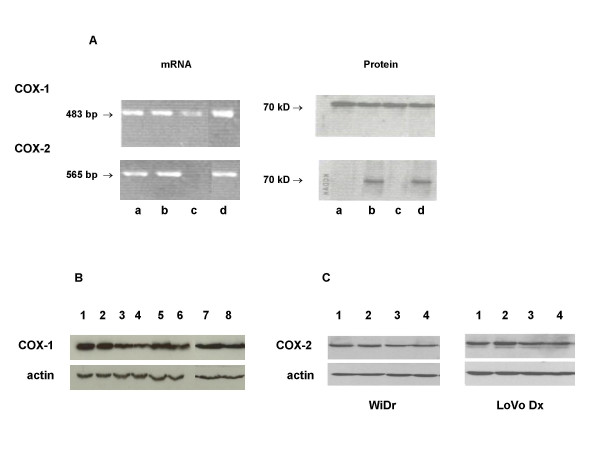
(A) COX-1 and COX-2 basal mRNA and protein expression in the different cell lines. Lane a, LoVo cells; lane b, WiDr cells; lane c, LRWZ cells; lane d, LoVo Dx. (B) COX-1 protein expression in the different cell lines. *LoVo*: lane 1, untreated cells; lane 2, cells exposed to 10 μM of NCX 4040 (24 h); *LoVo Dx*: lane 3, untreated cells; lane 4, cells exposed to 50 μM of NCX 4040 (24 h); *WiDr*: lane 5, untreated cells; lane 6, cells exposed to 50 μM of NCX 4040 (24 h); *LRWZ*: lane 7, untreated cells; lane 8, cells exposed to 50 μM of NCX 4040 (24 h). (C) COX-2 protein expression in WiDr and LoVo Dx cell lines after different exposure schemes to 10 μM of NCX 4040. Lane 1, untreated cells (24 h); lane 2, NCX 4040 (24 h); lane 3, untreated cells (24 h) + washout (24 h); lane 4, NCX 4040 (24 h) + washout (24 h).

All the cell lines used were positive for the presence of the isoenzymatic form COX-1, as evaluated by mRNA and protein expression (Fig. 5A). Furthermore, after a 24-h exposure to NCX 4040, the COX-1 expression level remained unchanged in all the cell lines (Fig. 5B). COX-2 mRNA and protein expression were both observed in WiDr and LoVo Dx cell lines, whereas neither was detected in LRWZ, and only mRNA was observed in LoVo cells. Furthermore, in WiDr and LoVo Dx cells, no change was observed in the COX-2 protein expression level after a 24-h exposure to NCX 4040 or a 24-h exposure followed by a 24-h washout (Fig. 5C).

**Figure 6:**
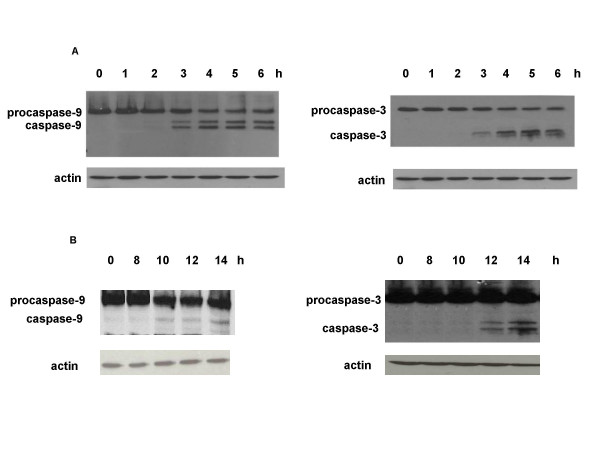
(A) Activation of caspases-9 and -3 after exposure times of 1, 2, 3, 4, 5 and 6 h to 10 μM of NCX 4040 in LoVo cells. (B) Activation of caspases-9 and -3 after exposure times of 8, 10, 12 and 14 h to 50 μM of NCX 4040 in LRWZ cells. An antibody for actin was used as loading control.

Although all 4 lines basally expressed the 2 caspases (data not shown), these proteases were activated only in LoVo and in LRWZ cells, where apoptosis was found. In particular, in LoVo cells caspase-3 and -9 activation was already evident after 3 h, whereas in LWRZ, activation took a longer time to occur (at least 10 h for caspase-9 and 12 h for caspase-3). The temporal activation in these 2 cell lines corresponds to the early or late induction of apoptosis (Fig. 6A,6B).

### In vivo studies

**Figure 7:**
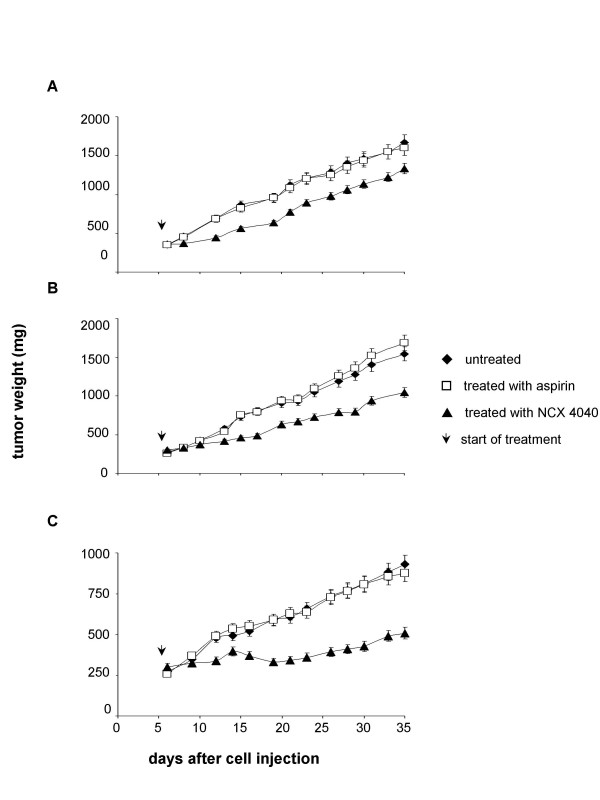
Antitumor efficacy in mice implanted with LoVo Dx (A), WiDr (B) and LoVo (C) cell lines. Each experimental group included 8 mice, each experiment was repeated at least three times, and representative independent experiments are reported. Experimental points represent means of 24 experiments (bars, SD). Arrows indicate the start of treatment.

The administration of aspirin in mice bearing all 3 cell lines did not influence the growth of tumors (Fig. 7A,7B,7C). In contrast, treatment with NCX 4040 was effective in the different cell lines employed, with a reduction of about 40% in the tumor mass (P < 0.001), evaluated at the nadir of the effect, compared to untreated and aspirin-treated groups. A difference in NCX 4040 sensitivity was also evident between the lines. Tumor regrowth was observed after 8 and 11 days for LoVo Dx and WiDr lines, respectively. Conversely, inhibition of the tumor mass for LoVo tumors was maintained from day 15 to day 30 following the injection of tumor cells and a tumor growth delay of 19 days was observed (P < 0.001). These results are in agreement with the *in vitro *data, which demonstrated that LoVo line is the most sensitive to NCX 4040 exposure.

## Discussion

Our study evaluated the antitumor activity of the novel NO-aspirin NCX 4040 *in vitro *on a panel of human colon cancer lines with different genetic profiles representing clinical tumor heterogeneity, and *in vivo *on xenografted immunosuppressed mice.

Neither aspirin nor the denitrated analog, NCX 4042, influenced the growth of any of the cell lines studied, whereas NCX 4040 produced an important cytocidal effect. These findings suggest a pivotal role of the -NO_2 _group in the mechanism of action underlying its *in vitro *activity. Extensive evidence indicates that nitric oxide is capable of initiating apoptotic cell death in some cell types as a consequence of DNA-induced damage [29,30], but the pathways involved are still not fully understood. In the present study, it was seen that NCX 4040 induced apoptosis in two (LoVo and LRWZ) of the four cell lines investigated, albeit with a modulated activity. In particular, we observed early apoptosis in LoVo cells starting from a 6-h drug exposure, with a peak after 20 h, as shown by the TUNEL assay. We found that apoptotic death was induced by the concomitant activation of caspases-9 and -3 a few hours after NCX 4040 exposure.

In LRWZ cells, a relevant apoptosis was also observed but after a longer exposure time and at higher NCX 4040 concentrations, after which the irreversibly damaged cells underwent apoptotic death through the activation of caspases-9 and -3.

Moreover, two NO donors were used to better clarify the role of the aspirin component. These compounds, at concentrations similar to those utilized for NCX 4040, showed a very modest cytotoxic activity (data not shown), confirming the importance of the aspirin component for the antineoplastic effectiveness of the NCX 4040 molelcule.

In addition, WiDr and LoVo Dx cell lines, which proved to be resistant to NCX 4040-induced apoptosis, were characterized by baseline expression of COX-2 protein. This finding, which is consistent with data recently published for breast and colorectal cancer cells, would seem to support the hypothesis that a high level of COX-2 protein protects cells from apoptosis, especially that induced by NO-compounds [31,32].

With the exception of LRWZ, all the lines evaluated in *in vitro *studies showed a high tumorigenic capacity, making it possible to evaluate *in vivo *the antitumor efficacy of NCX 4040. The long-term administration of this drug was well tolerated by the mice and reduced the tumor mass of LoVo, LoVo Dx and WiDr lines compared to untreated mice. Analysis of growth curves highlighted differences in the degree of drug sensitivity between cell lines. NCX 4040 was very effective in delaying tumor growth in LoVo tumors, whereas WiDr, and especially LoVo Dx lines, regrew after the end of treatment. These results confirm the *in vitro *data and seem to indicate that, as observed *in vitro*, the ability of NCX 4040 to induce apoptosis has a key role in the antitumor efficacy of this drug.

## Conclusions

In conclusion, NCX 4040 was effective in reducing the growth of several human colon cancer cell lines both *in vitro *and *in vivo*, whereas the parental compound aspirin showed no activity, which suggests that the NO-releasing derivate could prove useful in the clinical management of colon cancer, possibly in combination with conventional antineoplastic drugs.

## Abbreviations

COX, cyclooxygenase; NO, nitric oxide; NO-NSAID, nitric oxide-releasing nonsteroidal antiinflammatory drug releasing; NSAID, nonsteroidal antiinflammatory drug; SRB, sulforhodamine B; TUNEL, terminal uridine nick end labeling.

## Competing interests

Manlio Bolla is an employee of NicOx SA.

## Authors' contributions

AT was responsible for study design, data analysis, and drafting the manuscript. WZ, DA and MB participated in the study design and acted as scientific advisors. AT, PU, FF and MR performed the *in vitro *experiments. CL, MS and GZ also participated in the study design and carried out the *in vivo *experiments. All authors read and approved the final manuscript.
